# Pro-Cognitive Properties of the Immunomodulatory Polypeptide Complex, Yolkin, from Chicken Egg Yolk and Colostrum-Derived Substances: Analyses Based on Animal Model of Age-Related Cognitive Deficits

**DOI:** 10.1007/s00005-016-0392-z

**Published:** 2016-03-14

**Authors:** Marta Lemieszewska, Marta Jakubik-Witkowska, Bartłomiej Stańczykiewicz, Aleksandra Zambrowicz, Agnieszka Zabłocka, Antoni Polanowski, Tadeusz Trziszka, Joanna Rymaszewska

**Affiliations:** 1 Division of Consultation Psychiatry and Neuroscience, Department of Psychiatry, Wroclaw Medical University, 10 Pasteura Street, 50-367 Wroclaw, Poland; 2 Department of Animal Products Technology and Quality Management, Wroclaw University of Environmental and Life Sciences, 37 Chelmonskiego Str, 51-630 Wroclaw, Poland; 3 Department of Immunochemistry, Ludwik Hirszfeld Institute of Immunology and Experimental Therapy, Polish Academy of Sciences, 12 Weigla Str, 53-114 Wroclaw, Poland

**Keywords:** Immunomodulatory peptides, Chicken egg yolk, Colostrinin, Coloco, Learning, Memory, Dementia, Age-related disorders, Cognitive deficits

## Abstract

The study aimed to assess the effect of the polypeptide Y complex (Yolkin), isolated from chicken egg yolk, on behavioural and cognitive functions. It also aimed to compare this activity with colostrum-derived substances (Colostrinin, Coloco), which have a confirmed impact on learning and memory. In the study, the effect of Yolkin, administered to rats of different ages, who performed various tasks involving spatial and episodic memory, motor functions and exploratory behavior, was assessed. The experiment was carried out in rats which were 6 and 12 months old. Two different doses of the studied specimens based on previous comparative studies and two different routes of administration (oral and retroperitoneal) were used. A series of behavioural tests were carried out, including an open field test, a novel object recognition test and a Morris water maze. They were used to evaluate the impact of the studied specimen on improving locomotor function and exploratory behaviour, preventing their decline and assess the functioning of episodic and spatial memory in aging rats. The administration of Yolkin gave distinct effects compared to colostrum-derived substances, although confirmed its suggested pro-cognitive action. Therefore, it may be used to enhance cognitive functions and inhibit the progression of dementia in the course of neurodegenerative disorders.

## Introduction

Cognitive disorders affecting memory and learning abilities normally occur in the process of aging, mild cognitive impairment and in Alzheimer disease (Lindeboom and Weinstein 2004; Pepeu 2004). The use of animal models, including transgenic mice and rats, and sensitive tests allows a precise detection of key behavioural markers of progressive degenerative disorders occurring in the brain. It also enables an analysis of the efficacy of therapeutic agents (Do Carmo and Cuello 2013). Preclinical studies are most often carried out on rats due to their complex behaviour, easier learning of cognitive tasks and their lower levels of aggression compared to mice. Rats also cope better when carrying out complex instrumental tasks and exhibit less stress when placed in water (Iannaccone and Jacob 2009).

For nearly 75 years, studies have been carried out on substances delaying symptoms of brain ageing and neurodegenerative disorders. The anti-oxidative and anti-inflammatory properties (Xiao and Tundis 2013) of pituitary peptides (de Wied 1997; Lukas et al. 2012) and numerous natural bioactive substances of plant (such as coffee) or animal (such as marine crustacean) origin has been demonstrated on animal models (Shukitt-Hale et al. 2013). Milk is characterized as a product of animal origin with a high content of proline-rich protein precursors. Proline is an amino acid with a characteristic heterocyclic compound, which determines its high chemical activity, including its anti-oxidative capability (Camfield et al. 2011). The mammary gland secretions during lactation, especially of colostrum, are of particular interest in terms of their biological activity and potential health benefits. Colostrum contains peptides and proteins rich in proline, which support development and the immune system (Zimecki and Artym 2013).

The biochemical and immunological properties of colostrum and its components have been tested in both animal models and in vitro (Darewicz et al. 2011). A proline-rich polypeptide complex (PRP), later named Colostrinin (CLN), was isolated from ovine colostrum in 1974 (Janusz et al. 1974). Colostrinin was found to have anti-inflammatory and antioxidant properties based on cell culture and in vivo studies. It was also found to regulate growth as well as differentiation of lymphocytes and inhibit pathological conditions associated with β-amyloid aggregation (Boldogh and Kruzel 2008; Janusz and Zabłocka 2010). A nonapeptide, which is a component of the PRP complex, was also found to have anti-aggregating properties (Janusz et al. 2009). The CLN complex was also found to have strong neuroprotective activity inhibiting nerve cell apoptosis induced by the deposition of toxic amyloid (Douraghi-Zadeh et al. 2009; Schuster et al. 2005). Colostrinin improves learning and memory in rats, delays the progression of dementia and loss of long term memory in aging animals (Popik et al. 1999, 2001; Stewart and Banks 2006). Positive results of preliminary clinical trials on patients with Alzheimer disease, in which from a total of 15 patients receiving oral CLN at a dose of 100 μg, eight improved according to the Mini-Mental State Examination, while disease symptoms stabilized in the remaining seven patients (Leszek et al. 1999). These results were confirmed in multicenter double blind clinical studies performed on 105 patients (Bilikiewicz and Gauss 2004).

The discovery of procognitive and therapeutic properties of the CLN prompted the search for other substances with similar biochemical characteristics, which would also be widely available and contain an appropriate amount of biologically active and bioavailable ingredients. These substances should also be easy to identify, isolate and process pharmaceutically. Polypeptide complexes associated with immunoglobulin antibodies found in egg yolk are functionally equivalent to those found in colostrum (Kim et al. 2000; Tini et al. 2002). A polypeptide complex associated with a major avian immunoglobulin class IgY present in egg yolk, called Yolkin (Y), was found and isolated in the course of studies (Polanowski et al. 2012). Its similarities to CLN in terms of immunoregulatory properties, including stimulation of cytokine secretion, have been shown. These properties may also indicate a similarity between Yolkin and CLN in terms of the pro-cognitive activity (Kruzel et al. 2001). The present study aimed to assess the behavioural and cognitive effects of Yolkin—immunomodulatory peptide complex isolated from chicken egg yolk and to compare it with colostrum-derived substances—Colostrinin and recently separated Coloco-PRP (Polanowski et al. 2013, 2015).

## Materials and Methods

### Ethics Statement

Animals used in the study were provided by the animal house located at the Division of Pathomorphology at the Wroclaw Medical University. All experiments were conducted in accordance with the NIH Guide for the Care and Use of Laboratory Animals and approved by the 1st Local Committee for Experiments with the Use of Laboratory Animals, Wroclaw, Poland. Animals were killed by decapitation under anesthesia with ketamine (0.5 mL of 100 mg/kg body weight) at a dosage of 0.55 mL/100 g body weight.

### Subjects

Male Wistar rats (*n* = 120) at the age of 6 months and body weight ranged from 300 to 350 g were divided into two groups (*n* = 60), housed in pairs, in the standard medium-sized laboratory rat cages (ca. 45 × 35 × 20 cm). One group received the preparations in the form of intraperitoneal (i.p.) injections and the other in the form of an oral aqueous solution. Each group was divided into subgroups, receiving the following preparations diluted in 0.5 mL of vehicle (0.9 % NaCl) per rat: Yolkin at 100 μg/kg of body weight (Y_100_), Yolkin at 10 μg/kg of body weight (Y_10_), Colostrinin at 50 μg/kg of body weight (CLN), Coloco-PRP at 50 μg/kg of body weight (Col) and the control group, receiving either an i.p. 0.9 % NaCl (S) or an oral aqueous solution of bovine serum albumin (BSA) at 100 μg/kg of body weight. During the oral administration of ca. 100 mL of liquid per rat per day, the rats had to drink the bottle with the study solution, afterwards it was replaced with tap water. It was not intended to determine exact dosage, as the oral administration needs to have the nature of supplementation. Subgroups and preparations were matched randomly and blinded by experimenter’s assistant before the experiment, so the experimenter was blind to the treatment conditions.

### Preparations

**Fig. 1:**
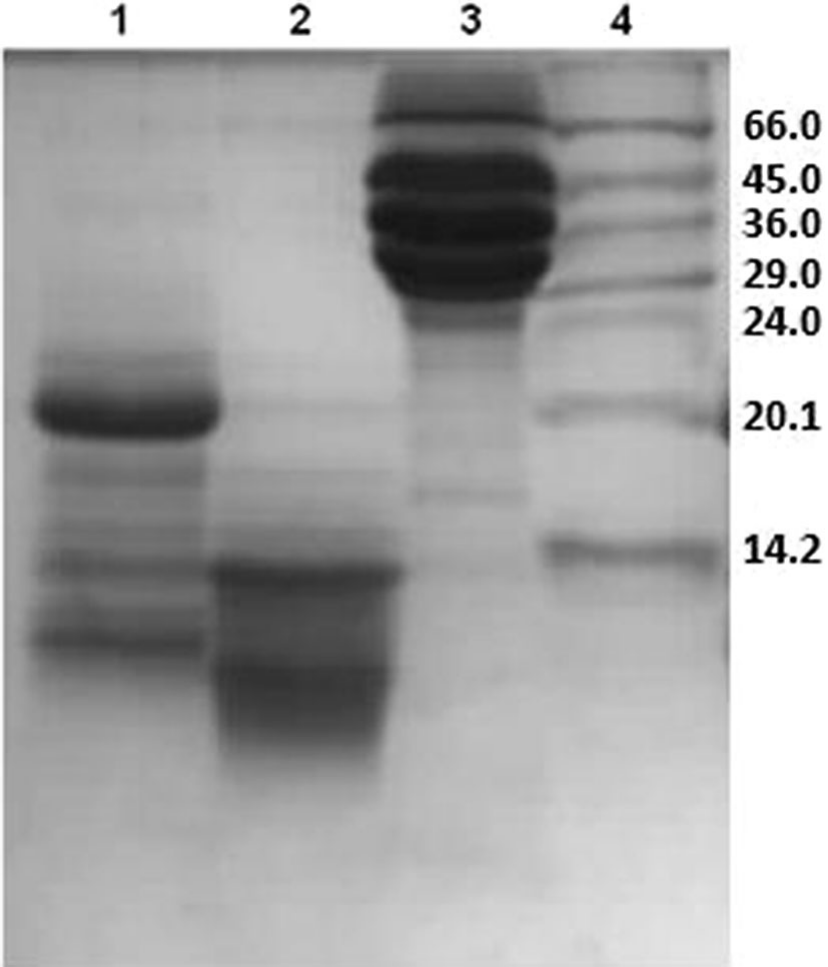
SDS-PAGE of the analysed preparations: Coloco-PRP (*1*), Colostrinin (*2*), Yolkin (*3*), molecular weight marker SDS-7;14–66 kDa (Sigma, Germany) (*4*)

Colostrinin and Coloco-PRP were obtained from bovine colostrum. The substances were isolated according to the method developed by Sokołowska et al. (2008) and Polanowski et al. (2015). Yolkin was isolated from chicken egg yolk using the method described by Polanowski et al. (2012, 2013). SDS-PAGE showed that CLN consists mainly of peptides with a molecular weight less than 14 kDa, Coloco-PRP contains peptides with a weight up to 20 kDa, while Yolkin is a complex composed of products of proteolytically cleaved C-terminal fragment of the vitellogenin II called YGP 40 (Yamamura et al. 1995) (Fig. 1). Their constituents have molecular weight ranging from over 1 to about 35 kDa with a clear predominance of peptides of molecular weight ranging from about 16–23 kDa.

### Experimental Schedule

**Fig. 2:**
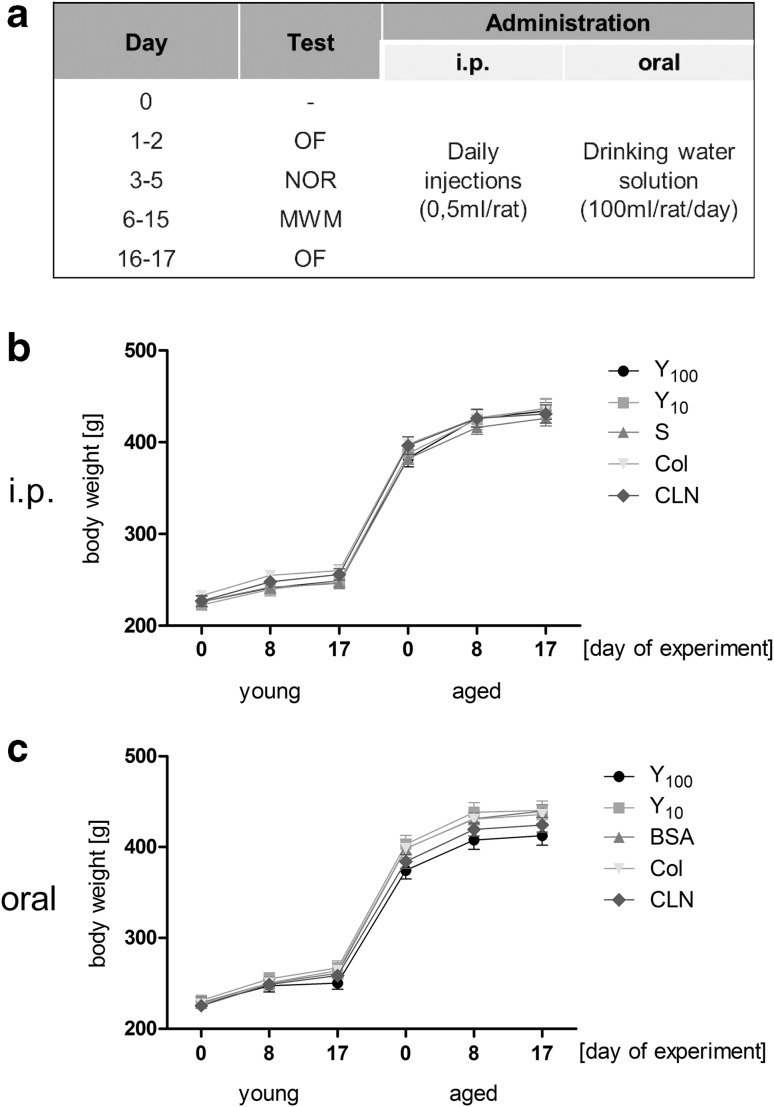
Experimental schedule [repeated for young (6 months old) and aged (12 months old) rats] (**a**) and body weight changes during the experiment: i.p. (**b**) and oral administration (**c**)

The rats were housed in pairs, in standard conditions (with the light phase on between 6:00 am and 6:00 pm at a temperature of 23 ± 1 °C), with ad libitum access to food and water. Administration of study specimens began day before the first testing day and ended the day before the final testing day (the preparations were administered for a total of 17 days). Detailed experimental schedule was presented on the Fig. 2a. Body weight was checked three times during each experimental series (on day 0, 8 and 17, results presented on Fig. 2b, c). All behavioural tests were carried out during the light phase. Approximately 1 week before the beginning of the experiment, all animals were familiarized with the experimenter (daily handling for ~1 min). The tests were carried out at an ambient temperature of 22–23 °C in dispersed light to limit the anxiety of the tested animals.

### Open Field Test

The open field (OF) test was carried out first, where the locomotor activity and exploratory behaviour were assessed, so that the equal groups of animals can be arranged. Rats were placed singly in a square (area: 1 m^2^, height: 0.5 m) box and were observed for 5 min. Two trials were carried out: at the beginning (day 0, t1) and at the end (day 17, t2) of experiment. The distance covered in the testing area, reflecting the rats’ activity, was measured and analyzed. Rats’ movement was monitored using a video camera with SMART software (PanLab, Spain).

### Novel Object Recognition

The novel object recognition (NOR) test evaluates the ability to recognize a new object in animal’s environment. It is based on a natural mechanism of initiating interest and novel object exploration—typical for rats and other rodents (Antunes and Biala 2012). The test was carried out 48 h after OF test, in the same testing box, thereby animals were already habituated to testing area. The actual test was composed of familiarization phase and the test phase. At first, two identical objects were placed in the test area. The animal was placed behind the objects, facing the opposite direction, and was then allowed to explore them for 5 min.

The proper test was carried out 24 h later. One of the objects from the previous phase and a second, new one (of different shape, texture and colour) were placed in the test area and the time of contact with each object was recorded. The exploration time for both objects in the familiarization and test phases were monitored and the novelty preference index of the new object compared to the familiar object was calculated using the equation *B*/(*A* + *B*), where *A* indicates % of time spent on exploring familiar object, and *B* indicates % of time spent on exploring novel object. Preference index value above 0.5 indicated that the rat showed interest in the new object for a longer period of time than with the object it was previously familiarized with. All calculations were based on the data recorded using SMART software (PanLab, Spain).

### Morris Water Maze

The Morris water maze (MWM) test was carried out according to the standard spatial learning protocol (Vorhees and Williams 2006). A 180 cm diameter tank was filled with dark-coloured water (to improve the recorded image contrast and increase animal tracking precision) at a temperature of 22 °C and to a depth of 30 cm. The tank was surrounded by high-contrast visual cues. A platform was located ~50 cm from the tank wall and submerged 1 cm below the water surface so the rat was not able to see it when dropped into the tank. The test consisted of an eight-trial acquisition training (learning phase) and one probe trial session (test phase). Four drop locations (N, W, S, E) were pseudo-randomized and each subject was given a 120-s trial of training per day. After finding the platform, the rat was allowed to rest on it for 5–10 s. If the rat failed to locate the platform within the given time, it was placed on it for 30 s by the experimenter, then it was dried and placed back in its cage.

In the probe trial, the platform was removed and the subject was allowed to swim for the 60 s to measure spatial learning. Swim paths were tracked and recorded by a video tracking system directly above the water tank and parameters were measured using SMART software (PanLab, Spain). The recording area was divided into quadrants (NE, SE, SW, NW) with separated ring-shaped target zone in SW quadrant. The learning phase path distance, probe trial distance, average distance swam in the target quadrant and the number of crossings of the target zone were calculated and analyzed.

### Statistics

Eight to ten animals were used in each group for statistical analysis. Intergroup effects of certain treatments and interactions between treatment and age of animals were assessed using two-way ANOVA with Bonferroni post hoc tests. Open field activity was analyzed using three-way mixed design ANOVA, including age, treatment (between-subject factors) and trial number (within-subject factor) interactions. All the results are presented as mean group values with the standard error of the mean (±SEM). The results were considered statistically significant when *p* ≤ 0.05 compared to the control group (animals receiving the placebo). The statistical analysis was carried out using the IBM SPSS Statistics 20 (IBM Corporation 2011) software, and the results were presented in the form of graphs using the GraphPad Prism 5 (GraphPad Software Inc. 2007) software.

## Results

### OF Test Results

**Fig. 3:**
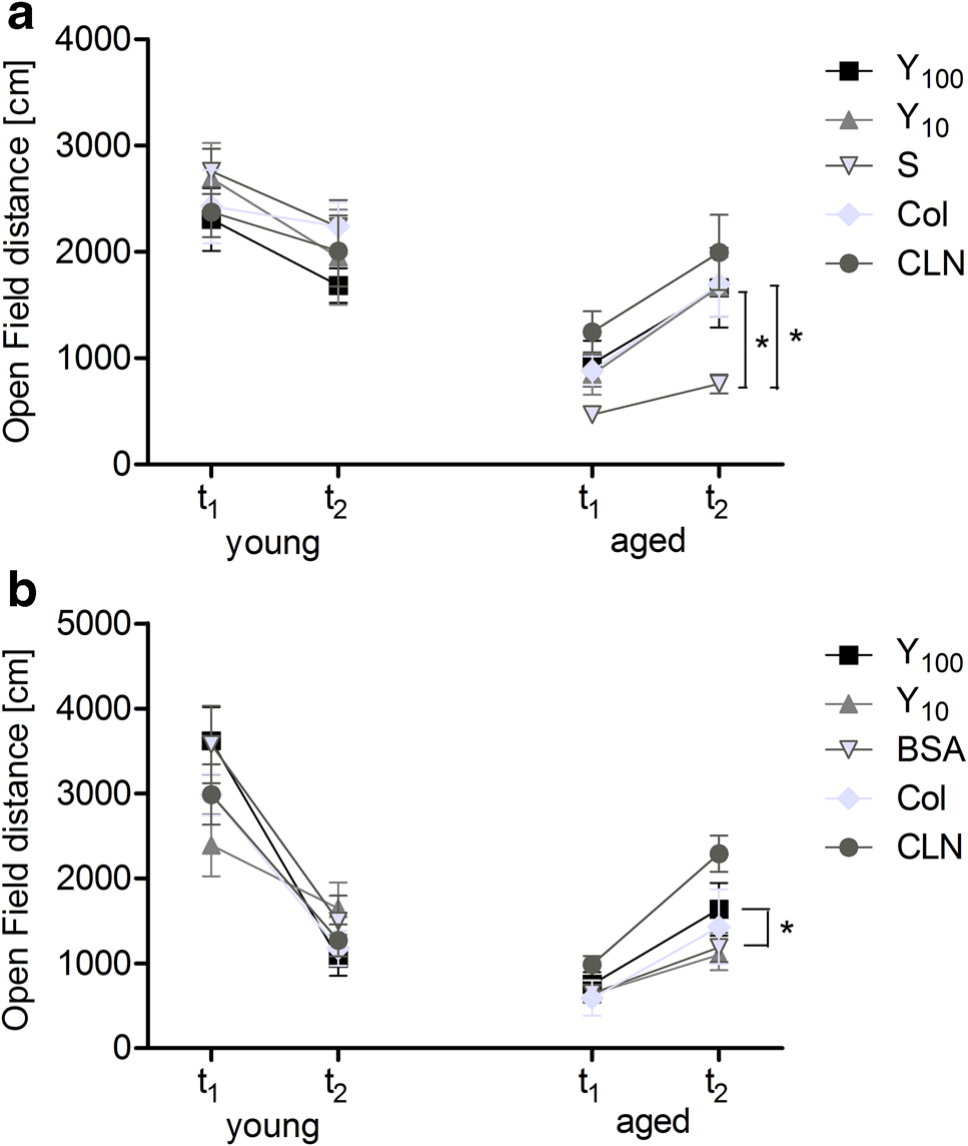
Open Field test. Results present the locomotor activity expressed as the distance covered by animals in groups treated intraperitoneally (**a**) or orally (**b**). *Asterisk* indicates a significant difference compared to the placebo group at *p* < 0.05

The interaction between age, treatment and trial in rats treated intraperitoneally did not reach statistical significance, but results in the oral group achieved significant three-way age × trial × treatment interaction (*F*_(4.177)_ = 2.720, *p* < 0.05). However, overall locomotor activity of aged rats treated intraperitoneally was slightly affected by both doses of Yolkin (independent *t* test results for Y_100_ vs S = 2.890 and Y_10_ vs S = 2.696, *p* < 0.05; Fig. 3a). In the group of rats treated orally, statistically significant effect was observed only with higher dose of Yolkin (Bonferroni posttest: Y_100_ vs BSA = 2.830, *p* < 0.05; Fig. 3b).

### NOR Test Results

**Fig. 4:**
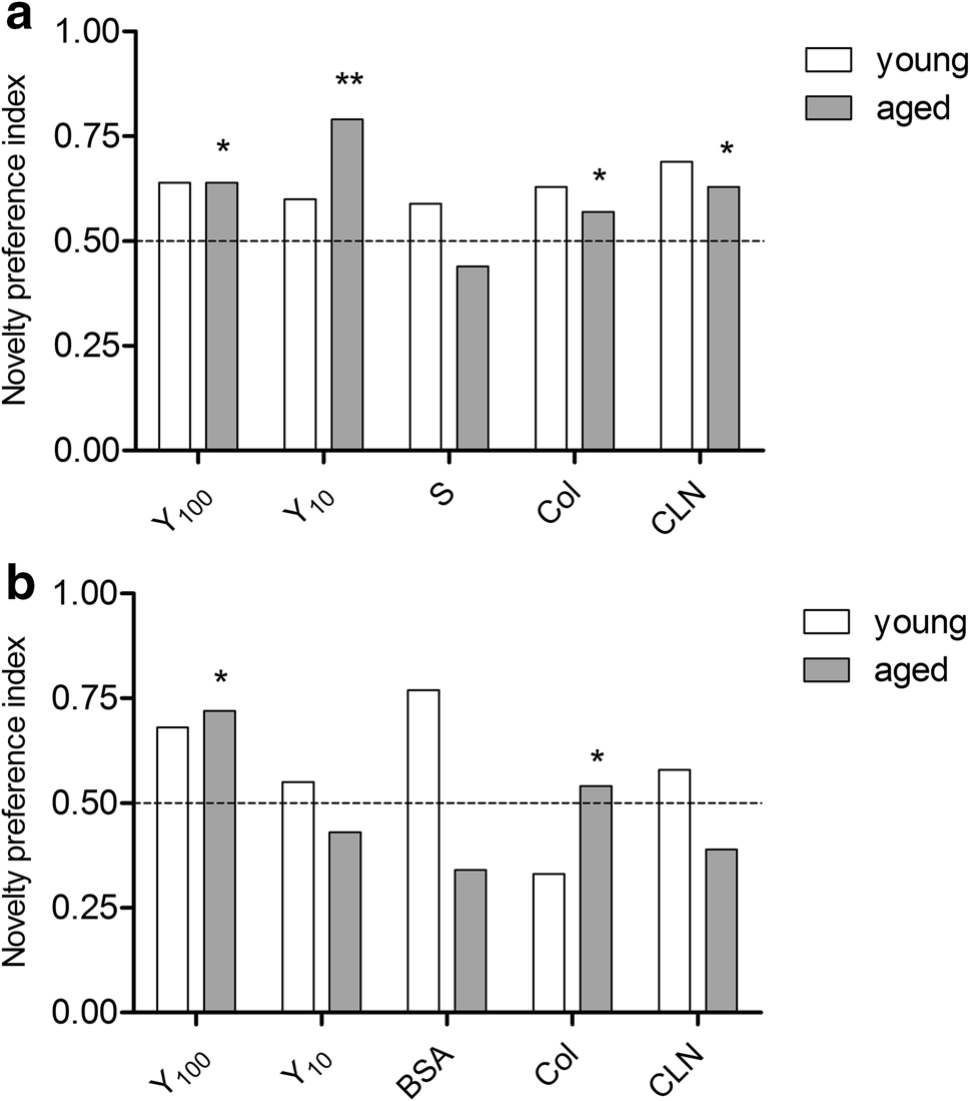
Novel Object Recognition results (test phase, T2). Preference to explore a novel object in the testing area expressed as the proportion of time of exploration of a new object (*B*) (during the test phase) compared to that of a known object (*A*) according to equation *B*/(*A* + *B*). Values below 0.5 indicate a lack of interest in the new object. Mean ± SEM in groups receiving each preparation/placebo, either intraperitoneally (**a**) or orally (**b**). *Asterisks* indicate a significant difference compared to the placebo group, according to Bonferroni posttest: * *p* < 0.05, ** *p* < 0.01, respectively for young (*white bars*) and old (*grey bars*) rats

In both age groups, all animals receiving the Yolkin (i.p. and oral administration) showed higher preference to the novel object in their surroundings compared to the control groups. The two-way interaction between age and treatment reached strong statistical significance in the i.p. administration group (*F*_(4.66)_ = 2.853, *p* = 0.032), with no conventional significance (*F*_(4.61)_ = 1.961, *p* = 0.114) for the oral administration group. The preference to the novel object was measured as time spent on exploring objects in relation to the animal’s general activity in both age groups. The percentage of permanence time at the baseline (T1) and test (T2) trial as well as calculated preference indexes were presented in the table (Table 1). The significant effect of treatment in aged rats was shown for both Yolkin doses in i.p. administration group (Bonferroni post-tests: Y_100_ vs S = 2.464, *p* < 0.05; Y_10_ vs S = 3.215, *p* < 0.01; Fig. 4a) and for higher dose of Yolkin in oral group (Bonferroni posttests: Y_100_ vs BSA = 3.317, *p* < 0.05; Fig. 4b).

**Table 1:** NOR test results

	Baseline (T1)			Test (T2)		
	Object 1	Object 2	Preference index	Familiar object	Novel object	Preference index
	*x*	*y*	*y*/(*x* + *y*)	*x*	*y*	*y*/(*x* + *y*)
i.p. young						
Y_100_	1.83	1.66	0.47	1.65	2.88	0.64
Y_10_	2.42	2.74	0.53	1.94	2.96	0.60
S	0.67	0.40	0.38	1.06	1.52	0.59
Col	1.34	1.44	0.52	1.37	2.36	0.63
CLN	2.39	2.67	0.53	1.32	2.90	0.69
i.p. aged						
Y_100_	2.47	1.65	0.40	2.12	3.77	0.64*
Y_10_	3.02	2.32	0.43	1.24	4.73	0.79**
S	1.37	1.07	0.44	0.97	0.76	0.44
Col	0.28	0.29	0.51	4.65	6.17	0.57*
CLN	3.85	4.58	0.54	4.13	6.91	0.63*
Oral young						
Y_100_	1.10	1.52	0.58	0.75	1.62	0.68
Y_10_	1.31	1.00	0.43	1.37	1.70	0.55
BSA	0.93	1.36	0.59	0.81	2.68	0.77
Col	2.00	2.07	0.51	1.44	0.71	0.33
CLN	1.39	1.80	0.56	1.32	1.86	0.58
Oral aged						
Y_100_	4.56	4.93	0.52	4.23	10.65	0.72*
Y_10_	1.27	0.98	0.44	1.45	1.09	0.43
BSA	2.54	2.27	0.47	14.13	7.40	0.34
Col	1.21	0.57	0.32	2.24	2.60	0.54*
CLN	6.82	1.98	0.23	9.08	5.80	0.39

Each object value represents the mean permanence time (percentage of total exploration time) and the value of preference index was calculated according to equations provided. *Asterisks* indicate a significant difference compared to the placebo group, according to Bonferroni posttest * p < 0.05, ** p < 0.01

### MWM Results

**Fig. 5:**
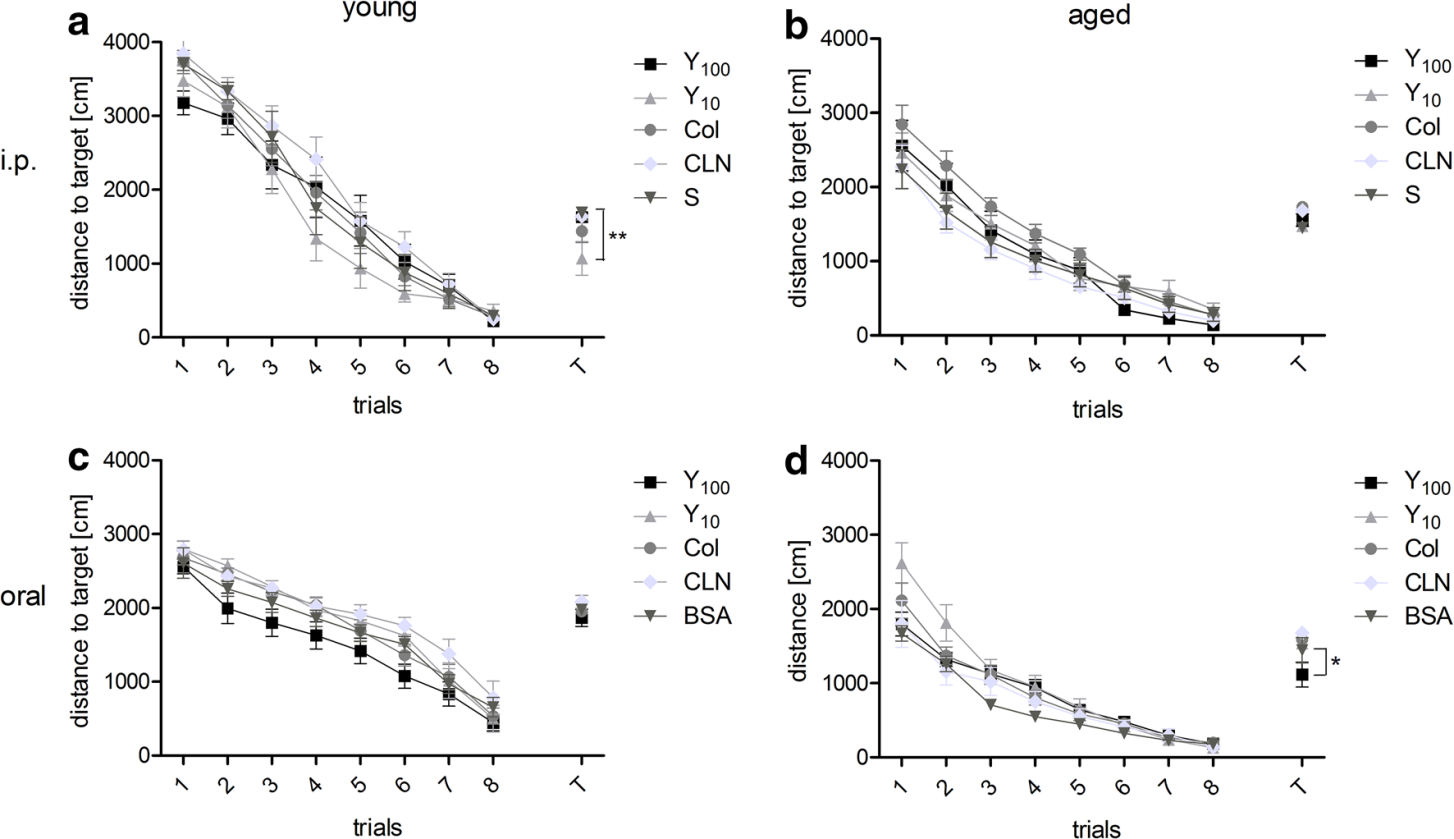
Results of the Morris water maze. The graphs represent the distance covered to reach the platform during the eight training trials (learning phase) and to make the first entrance to the target zone on the probe trial (T). **a**, **b** i.p. administration, **c**, **d** oral administration. *Asterisks* indicate a significant difference compared to the placebo group, according to Bonferroni posttest: * *p* < 0.05, ** *p* < 0.01

As presented in Fig. 5, the results obtained from eight training trials showed no significant differences between groups treated with preparations and placebo, regardless of the way of administration. However, in the probe trial, some distinct effects of Yolkin treatment can be observed. Strong age *x* treatment interaction was shown significant in two-way ANOVA only for i.p. administration group (F_(4.79)_ = 3.420, *p* = 0.013). Young rats receiving lower dose of Yolkin covered statistically shorter distance to target zone, than other groups (Bonferroni posttest: Y_10_ vs S = 3.342, *p* < 0.01; Fig. 5a). In the group of aged rats, statistically shorter distance was covered by those rats, which received higher dose of Yolkin in drinking water (Bonferroni posttest: Y_100_ vs BSA = 3.108, *p* < 0.05; Fig. 5d).

**Fig. 6:**
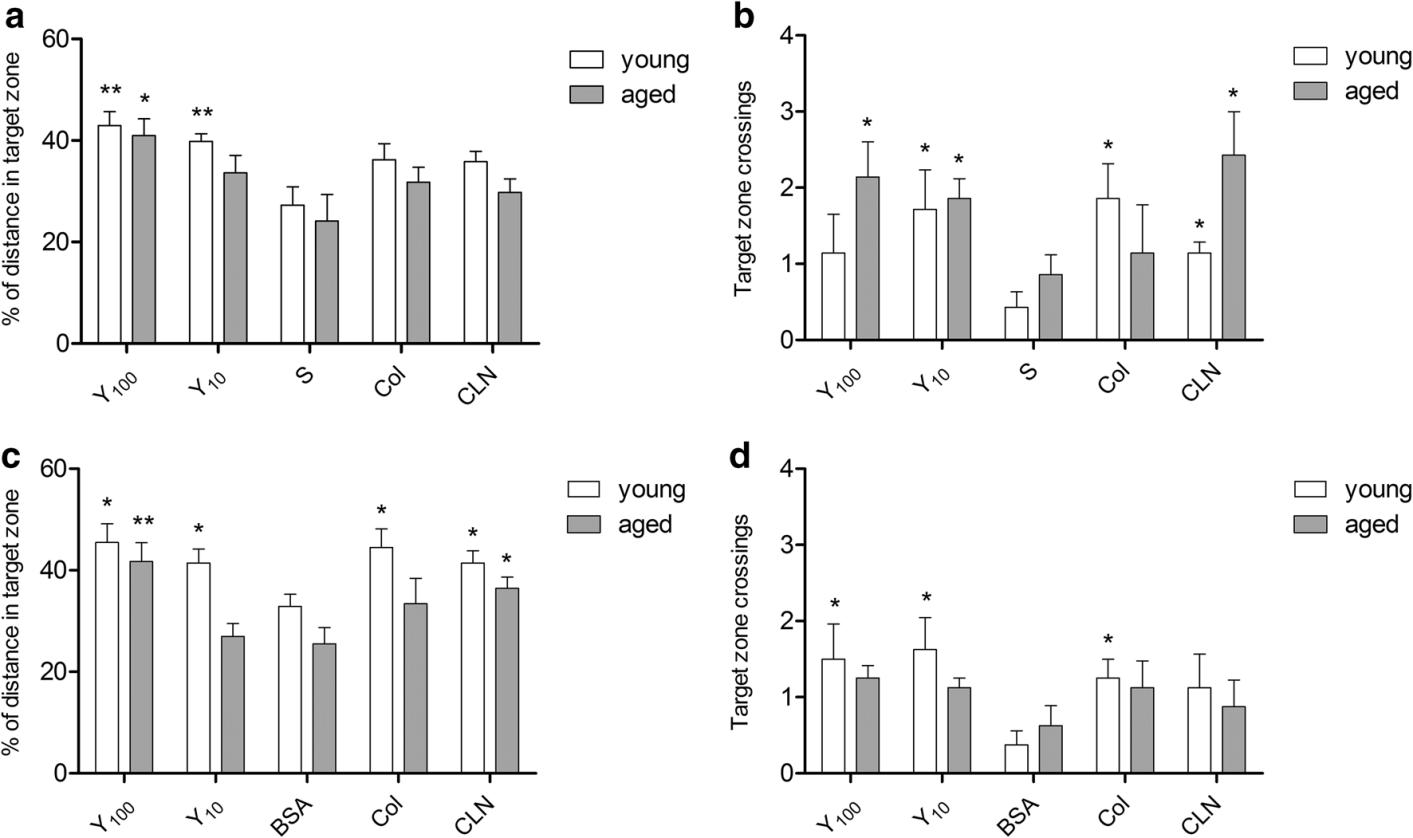
Detailed results of the Morris water maze (probe trial). The graphs represent the distance travelled in the target zone (SW) during the probe trial, expressed as percentage of the total distance covered (**a**, **c**) and the number of times the animal crossed through the target zone (**b**, **d**). **a**, **b** i.p. administration, **c**, **d** oral administration. All results are mean ± SEM. *Asterisks* indicate a significant difference compared to the placebo group, according to Bonferroni posttest: * *p* < 0.05, ** *p* < 0.01, respectively for young (*white bars*) and old (*grey bars*) rats

An increase in the distance the rats swam in the target quadrant and higher frequency of swimming into the platform ring zone indicated the effect of the Yolkin, CLN and Coloco-PRP treatment on spatial memory. These increases were significant for young rats at both Yolkin doses given both intraperitoneally (Bonferroni posttests: Y_100_ vs S = 3.430, Y_10_ vs S = 3.204, *p* < 0.01; Fig. 6a) and orally (Bonferroni posttests: Y_100_ vs BSA = 2.852, Y_10_ vs BSA = 2.358, *p* < 0.05; Fig. 6c). Statistically significant increase in the distance swam by the aged subjects were obtained only at higher doses of Yolkin (Bonferroni posttests: Y_100_ vs S = 2.721, *p* < 0.05, Fig. 6a; Y_100_ vs BSA = 3.339, *p* < 0.01; Fig. 6c). The number of crossings of the target zone were increased regardless of Yolkin dose, with the significant effects of i.p. administration in aged rats (Bonferroni posttests: Y_100_ vs S = 2.435, Y_10_ vs S = 2.711, *p* < 0.05; Fig. 6b) and oral administration in young rats (Bonferroni posttests: Y_100_ vs BSA = 2.260, Y_10_ vs BSA = 2.729, *p* < 0.05; Fig. 6d). However, significant differences was shown separately in both age groups, two-way ANOVA statistics did not show any significant interaction between age and treatment in the MWM test.

## Discussion

In the present study, both i.p. and oral administration of Yolkin derived from chicken egg yolk influenced the behavior and cognitive functions in young and aged rats. Yolkin primarily led to mitigating behavioral symptoms of aging and supported cognitive learning and memory in rats from both age groups. A distinct, stronger effect of Yolkin treatment, when administered intraperitoneally was seen both in young and aged rats, whereas an oral administration of Yolkin, particularly at the lower dose, turned out to be less effective in older rats. Neither i.p. nor oral administration significantly affected animals’ body weight gain during treatments.

The results previously obtained showed a pro-cognitive potential of Yolkin, in particular its effect on improving spatial and episodic memory given its immunoregulatory properties (Jakubik et al. 2013). Yolkin mitigated behavioural symptoms of aging and supported cognitive functions, learning and memory in young and old rats. Yolkin was found to act differently (these differences were statistically significant) in young and old rats depending on the route and dosage of administration. It showed some distinct effects in young and old rats when administered intraperitoneally, independent of the dose. When administered orally, it proved to be more effective at a higher dose. The effect of motivation and cognitive processing on the results obtained in these tests depending on the animals’ age could be assessed, since behavioral tests that examined various aspects of memory were applied. The results were more dispersed in groups of rats receiving preparations orally than intraperitoneally. Since there was a significant time interval between administration and carrying out the tests, the effect of the route of administration on the results was minimalized.

Current literature often indicates a biphasic response to pro-cognitive agents, which is manifested by improved cognitive abilities when receiving low doses and a worsening of behavioural test results on receiving higher doses. The biphasic activity of Colostrinin was shown in a study on 13 months old rats that received 4 μg of CLN intraperitoneally. This dose led to an improvement in spatial memory in the MWM, whereas, a fivefold higher dose led to its decrease (Popik et al. 1999). In this study, a worsening of effects was not observed at a Yolkin dose of 100 μg/kg. Moreover, the rats achieved better results in the MWM when receiving 100 μg/kg than 10 μg/kg. On the other hand, better NOR test results were achieved at lower Yolkin and Colostrinin doses (compared to the placebo group). This may be a proof for a phasic mechanism of action of procognitive agents.

From another point of view, the discrepancy in the results of animals from the same age group may be associated with the type of behavioural test used. Different tests involve a particular type of memory and require the subject’s motivation to perform a certain cognitive task. In the group of young rats, the results obtained from the OF test show the effect of habituation to the testing area, i.e., the animals remembered the area and the intensity of exploration in the second trial was much lower than activity presented at the first trial. Although, in the group of older rats the exploratory behaviour were slightly different. Results from the second trial revealed increased locomotor activity, which mostly can be the apparent psychostimulatory effect of treatment, but it indicates some contribution to restore age-related deficits during the treatment.

Apparent effects of Yolkin treatment, similar to colostrum-derived preparations was observed in the NOR test. Animals’ curiosity plays a key role in the object recognition test, when a new object is placed in the field of vision (Antunes and Biala 2012). However, object recognition memory in the experiment was maintained for a much shorter period of time than the memory used in the water maze test and it is easier to detect differences in the motivation to explore the objects, which was naturally greater in young animals than older ones. Therefore, NOR test seemed to be less specific in assessing the effectiveness of the pro-cognitive agents in older rats. The water maze test involves motivation mechanisms associated with the animal’s desire to leave the water as quickly as possible (D’Hooge and De Deyn 2001). It also encourages the animal to use previously trained behaviour and established spatial reference directions (Luparini et al. 2000). Better results obtained by some older rats may indicate their increased motivation to leave the water, although spatial reference directions established during the training phase most likely had affected the results. Therefore, the specificity of this test seems sufficient to correctly interpret the effect of the studied specimens on memory functioning.

Weaker effects of Coloco-PRP compared to Yolkin were observed in older rats based on the NOR results and the frequency of target zone crossing. Coloco-PRP and Colostrinin are isolated using various methods, which alter the peptide content, and, in effect, have an impact on the intensity of the pro-cognitive action. There are known literature reports presenting opposing effects of peptides with varying chemical compositions on memory and learning performance. Some studies on a colostrum-derived nonapeptide (Colostral-Val nonapeptide), showed it to have different effects to Colostrinin. It hampered the replacement of old memory traces with new ones and enabled memory retrieval, while Colostrinin enabled faster learning and a more efficient formation of new memory traces (Popik et al. 1999, 2001).

The results obtained for Coloco-PRP do not clearly indicate whether it reduces the efficiency of memory retrieval in older rats since worse results occurred only in terms of the number of target zone crossings. The remaining parameters did not differ. Therefore, it seems unlikely that Coloco-PRP could interchangeably lead to a worsening of spatial memory and NOR. Also, Coloco-PRP did not impair learning abilities of the rats during the training phase in the water maze.

The applied experimental model confirmed the efficacy of Yolkin in improving spatial and episodic memory in older rats compared to colostrum-derived preparations. NOR object recognition memory of the older rats remained at the level of the young rats. Spatial memory improved significantly in older rats following i.p. administration of Yolkin (particularly at higher dose), while it remained at the level of the young rats when Yolkin was administered orally.

In the development of neurodegenerative diseases the main risk factor is aging which, in combination with genetic and environmental factors leads to the manifestation of the illness. At the cellular level, in neurodegeneration, oxidative stress, mitochondrial dysfunction, overproduction of pro-inflammatory cytokines and decreased level of neurotrophic factors are included. There is some evidence indicating that brain-derived neurotrophic factor (BDNF) and interleukin 6 (IL-6) play an important role in the control of memory and learning (Allen et al. 2013; Balschun et al. 2004; Cunha et al. 2010; Leal et al. 2014; Lu et al. 2014). For survival, morphological/biochemical differentiation and regeneration of cultured sympathetic and sensory neurons, retinal ganglion cells as well as PC12 cells IL-6 is required (März et al. 1999; Oh et al. 2010; Perigolo-Vicente et al. 2013). The literature data also relates IL-6 to BDNF expression in neurons (Bartkowska et al. 2010). Reduced expression of neurotrophic factors can lead to dysregulation of neuron function and neuronal death. Therefore the upregulation of the level of neuroprotective factors is one of the key manners to control the nervous system function. It can be promising in the therapy of neurodegenerative disease in which the decreased level of trophic factors is observed. Natural origin regulatory substances such as Yolkin can be promising for therapeutic intervention, probably also in the early stages of dementia.

The mechanism of action of Yolkin is still under investigation. The recently obtained results indicate that Yolkin stimulates human whole blood cells to release cytokines: IL-6 and IL-10, shows the ability to modulate the nitric oxide (NO) production by mouse macrophages and protects against lipid peroxidation (Zabłocka et al. 2014). Also, Yolkin stimulates neuronal cells to produce and release the mature form of BDNF (the latest unpublished data). Based on the results obtained up to now we hypothesized that the neuroprotective effects of Yolkin may be associated with its effects on cells of the central nervous system (CNS) and also peripheral blood cells flowing to the brain through the damaged blood–brain barrier (BBB). These cells are stimulated to the production and secretion of substances with neuroprotective or/and regulatory effect on neurons.

It was known that NO released in physiological concentration plays an important role as an neurotransmitter. Despite of pivotal role of NO under physiological condition its overproduction can critically contribute to inflammation and autoimmune diseases. Therefore the ability of Yolkin to modulate the NO production has an important therapeutic meaning in the treatment of neurodegenerative disorders and can participate in the improvement of memory. IL-10 is a cytokine with the pleiotropic immunomodulatory effects. It downregulates the expression of pro-inflammatory factors, especially pro-inflammatory cytokines like interferon γ or tumor necrosis factor-α. The decreased level of IL-10 is observed in neurodegenerative processes. Demonstrated ability of Yolkin to stimulate of IL-10 speaks for its anti-inflammatory action. BDNF plays an important role in neuroprotection and in the control of the CNS functions. The physiological role of IL-6 in the CNS development and function is still under consideration (Gruol and Nelson 1997; Lessmann et al. 2003; Skaper 2008). The main source of IL-6 and BDNF in the CNS can be astrocytes and also blood lymphocytes which are able to infiltrate to the CNS by damaged BBB.

In conclusion, the presented study shows strong procognitive capabilities of a chicken egg-yolk derived preparation known as Yolkin. Some age-related cognitive deficits observed within the control groups of rats at an older age seemed to be restored during the Yolkin treatment, i.e., increased recognition of the novel object in the testing area or higher effectiveness in localizing the target zone in the MWM. The applied behavioural animal model of age-related cognitive dysfunctions has revealed the efficacy of Yolkin in improving spatial and episodic memory, due to the neuroprotective and immunomodulatory properties, in parallel with Colostrinin and Coloco-PRP. In the study, we also referred to current research on mechanism of action of Yolkin and colostrum-derived substances. Regarding the latest findings, we conclude that Yolkin may very likely present the remarkable solution for the prevention and treatment of aging-related neurodegenerative disorders.
